# Planetary sources of bio-essential nutrients on a prebiotic world

**DOI:** 10.1098/rstb.2024.0288

**Published:** 2025-10-02

**Authors:** Toni Galloway, Abu Saeed Baidya, Claire Cousins, Eva E. Stüeken

**Affiliations:** ^1^ School of Earth and Environmental Sciences, University of St Andrews, St Andrews KY16 9TS, UK

**Keywords:** origin of life, abiotic nitrogen reduction, reactive phosphorus, transition metals, hot springs, hydrothermal vents

## Abstract

Multiple bio-essential elements such as nitrogen and phosphorus are found in all forms of life discovered thus far. In addition to these major elements, many transition metals have been proposed as crucial components for prebiotic and early cellular processes. These elements must, therefore, have been available during the origin of life; however, the processes that mobilized them on the prebiotic Earth likely differed from today. We provide a review and discussion on planetary processes that influenced the availability of bio-essential elements on early Earth and propose the most likely environments to host key prebiotic reactions and perhaps primitive life, based on their supply of nitrogen, phosphorus and transition metals. In particular, terrestrial acidic hot spring systems and deep-sea hydrothermal vents may have created ideal conditions through abiotic nitrogen reduction, dissolution of reactive phosphorus species and leaching of siderophilic transition metals from igneous bedrocks. This concept has implications far beyond Hadean Earth, as similar geothermal systems once existed on the surface of Noachian Mars, providing a comparable suite of elements, suggesting that crucial steps towards an independent origin of life may have unfolded on Mars and perhaps other terrestrial planets.

This article is part of the theme issue ‘Origins of life: the possible and the actual’.

## 1. Introduction

All life as we know it is composed of carbon (C), hydrogen (H), nitrogen (N), oxygen (O), phosphorus (P) and sulfur (S), as well as a suite of transition metals and metalloids, which are used for redistributing electrons and catalysing reactions in metabolic pathways. While the relative proportions of these basic ingredients for life’s recipe have likely evolved over Earth’s history in response to crustal evolution [1,2], ocean oxygenation [3,4] and changing biospheric demand [5], the wide distribution of these elements across the biosphere [6–8] suggests that many of them have played an important role in biochemistry since the origin of life. In fact, it is likely that the availability of many of these elements on the Hadean Earth dictated the pace, location and trajectory of prebiotic chemistry and would do the same for an independent origin of life on other worlds. We cannot be certain exactly which elements were required in prebiotic reaction networks on Earth or elsewhere, but at some point, life on Earth emerged with a demand for C, H, N, O, P and S and a variety of metals. Understanding the roots of biology, therefore, requires knowledge about planetary processes that control the supply of key elements in a soluble and reactive form. In this contribution, we review mechanisms that can generate reactive nitrogen, phosphorus, and transition metals under Hadean conditions and possibly on other planets such as early Mars.

## 2. Prebiotic nitrogen fluxes

Carbon-based life on Earth uses N, because the C–N bond is weakly polar, which makes N-bearing organic molecules more versatile in their reactive properties and three-dimensional structure. It is, therefore, conceivable that carbon-based life on another planet would also require nitrogen in its biochemistry [9]. Organic-bound N occurs mostly in the form of amines (R-NH_2_), where N has a redox state of −3. However, the most abundant form of N at Earth’s surface is and likely always has been relatively unreactive atmospheric N_2_ gas [10]. Biological N_2_ fixation constitutes the dominant flux of nitrogen into the biosphere today (*ca* 1.1 × 10^13^ mol yr^−1^ in the marine realm, [11]). Prior to the emergence of this metabolism, an abiotic mechanism may have generated ammonium for incorporation into prebiotic reaction networks, possibly via nitrogen oxides or cyanide, which were later converted into ammonium.

The most significant abiotic source of reactive nitrogen in the form of nitrogen oxides today is lightning. Within the plasma channel of a lightning strike, all atmospheric gases are atomized and ionized, and these ions can recombine into new molecules during cooling. N-atoms derived from N_2_ thus combine with O-atoms derived from O_2_, CO_2_ or H_2_O to form NO, which can in turn undergo further reactions to form nitrite and nitrate. This process delivers *ca* 3.5 × 10^11^ mol yr^−1^ of reactive nitrogen oxides to Earth’s surface today [12]. On the early Earth, when atmospheric O_2_ was vanishingly low (<10^–6^ bar), but CO_2_ was significantly higher than today (0.01−0.1 bar) [13], the products from this reaction were overall less oxidized (i.e. less efficient conversion of NO to nitrate) but still yielding a total flux of *ca* 10^9^−10^10^ mol yr^−1^ [14–16]. Additional nitrogen oxides may have been generated during volcanic eruptions and impact shock events, with estimated fluxes of 10^9^−10^11^ and 10^10^−10^11^ mol yr^−1^, respectively [17,18]. Within the ferruginous ocean, these nitrogen oxides may have undergone spontaneous reaction with dissolved and mineral-bound Fe^2+^ to form ammonium [19], though with possible leakage of NO and N_2_O gas back into the atmosphere [20]. Abiotic nitrate reduction can proceed at room temperature [19,21] and under hydrothermal conditions [22]. Some model calculations suggest that the prebiotic ocean may have accumulated <1 µM nitrate [23] and 10−100 µM ammonium [24] (for the latter, assuming pH 6−7 and a total source flux of fixed N of 10^11^ mol yr^−1^ that is converted to ammonium [24, tbl. 2]). Others have derived a few hundred millimolar ammonium in the ocean, though assuming no conversion of ${\text{NH}}_{4}^{†}$ to NH_3_ [25].

Hydrothermal settings have the additional capability of reducing N_2_ directly to ammonium [22,26]. Estimated ammonium fluxes resulting from this reaction range from 10^9^ to 10^12^ mol yr^−1^, depending on model assumptions for the supply of reaction catalysts, such as iron sulfides, magnetite or native iron−nickel. Brandes *et al.* [22] found that the reaction is favoured at low water activity and elevated pressure and temperature (>500 bar (5 × 10^7^ Pa), >500°C). Hence abiotic conversion of N_2_ to ammonium is probably most efficient in the deep crust, but given the right mixture of catalysts, it may proceed at significant rates in the upper crust in active hydrothermal systems [26]. Importantly, modern hydrothermal fluids can be highly enriched in ammonium to millimolar levels; however, these high concentrations are typically derived from remobilization of organic-bound ammonium [27] and therefore cannot provide a prebiotic benchmark. Igneous crustal rocks can be enriched in ammonium to up to *ca* 20 µg g^−1^ through magmatic differentiation [28], which may contribute ammonium to hydrothermal and weathering fluids at a low rate (10^8^–10^9^ mol yr^−1^) [29].

Besides ammonium, another potentially important class of N-bearing molecules for prebiotic chemistry may have been nitriles (organic molecules with –C≡N groups), including hydrogen cyanide (HCN), which has been invoked as a precursor in the lead up to the RNA world [30]. Models suggest that HCN could have formed in Earth’s early atmosphere through a combination of photochemical reactions, yielding a flux of *ca* 3 × 10^9^ mol yr^−1^ [31]. Larger amounts of nitriles (*ca* 10^11^ mol yr^−1^) could have been produced in the aftermath of meteorite impacts, which may have rendered Earth’s atmosphere highly reducing for a few million years [32]. During those episodes, additional nitriles could have formed in hydrothermal settings from reactions between fluids and impact-generated graphite [33]. A similar reaction between ammonium and reduced carbon compounds can generate HCN under crustal conditions independent from impact events [34].

**Figure 1.**
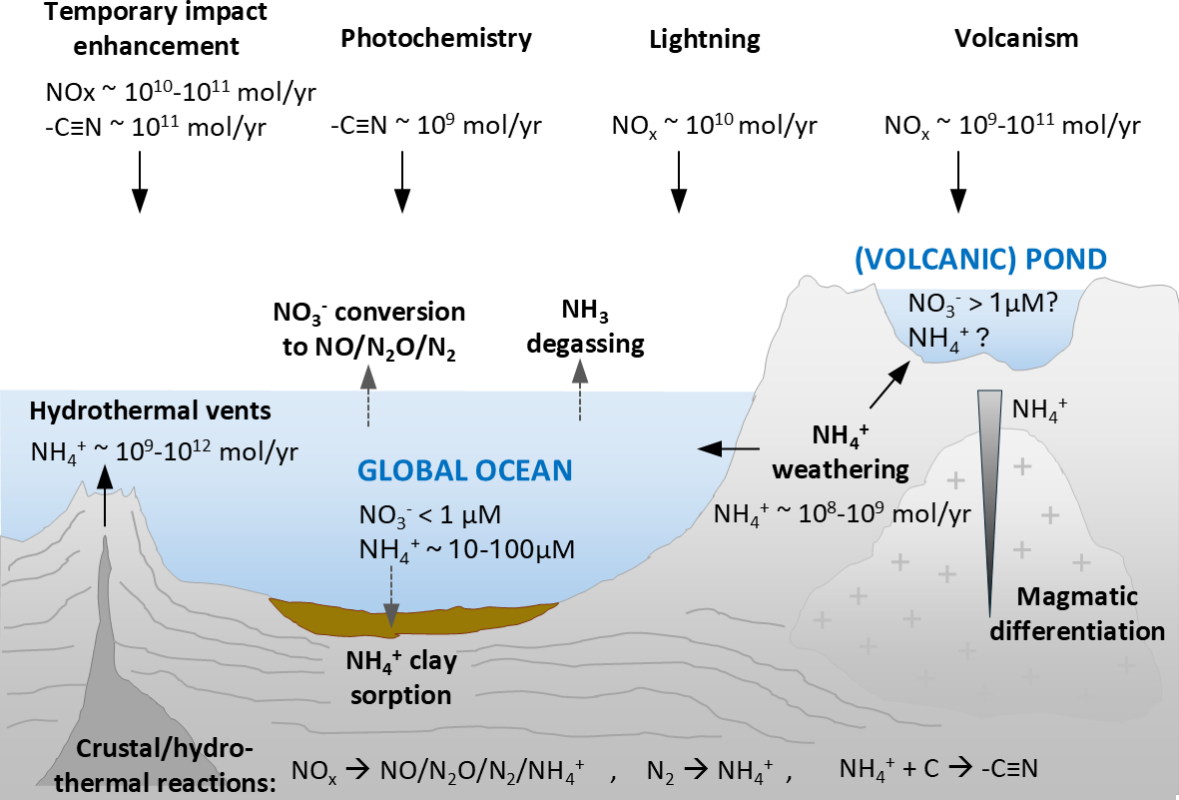
Schematic of abiotic nitrogen sources and sinks on the prebiotic Hadean Earth. See text for references. Note that the constraints provided for terrestrial ponds are not limited to volcanic settings but may apply there as well, aligning these sites with hot springs and volcanic ponds discussed in the context of P and transition metals (see §§ 3 and 4)*.*

In conclusion, a variety of mechanisms have been identified that likely generated reactive forms of nitrogen on the prebiotic Earth (figure 1). Importantly, ammonium is stable under anoxic conditions in seawater, with the major sinks being outgassing of NH_3_ and adsorption of NH_4_^+^ onto clay minerals [24]. Hence, marine settings may have been moderately ammonium-rich (10–100 µM; figure 1), creating opportunities for the formation of organic amines in hydrothermal settings [35]. Higher concentrations of dissolved nitrogen species could have been achieved in terrestrial ponds under favourable drainage conditions [23]. Impacts may have temporarily enhanced the formation of nitriles [32]; however, it needs to be considered that weathering of impact debris likely increased ocean pH [36], which would have enhanced NH_3_ degassing and lowered dissolved ammonium levels to a few micromolar [24]. Regardless, it is conceivable that the early biosphere continued to be supplied by abiotic N-sources until productivity increased and biological N_2_ fixation evolved.

## 3. Phosphate solubility, reduction and polymerization

Phosphorus is a key element for modern biological systems as it is required for phospholipids in cell walls, for energy production (e.g. ATP, phosphoenolpyruvate and creatinephosphate), and for information storage in DNA and RNA. It is, therefore, considered that P was and has always been crucial for biology since life originated on our planet [37,38]. Genomic studies confirm life’s dependency on P since the early stage of evolution [39], but geochemical modelling suggests that phosphate might have been the ultimate limiting nutrient for the biosphere throughout geological time [4]. Unlike N, P requires dissolution of rocks in order to be released in sufficient quantity into water before it can be incorporated into biomolecules [40]. Most P on our planet exists as phosphate (P(V)) with an oxidation state of +5, which is locked in sparsely soluble minerals such as apatite and vivianite [41,42]. Furthermore, P(V) is only weakly reactive to organic molecules. This low-solubility and low-reactivity have led to the coining of the term ‘P-problem’ for the origin of life [40].

**Figure 2.**
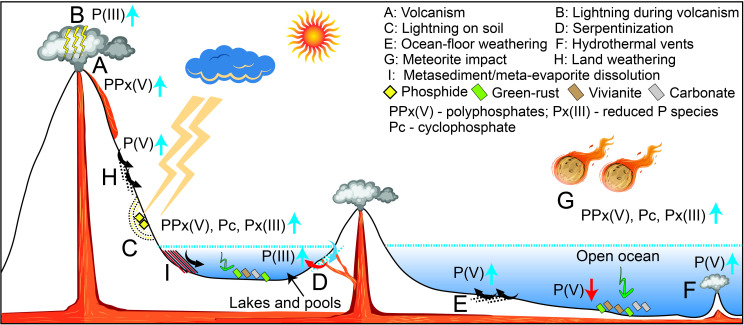
Sources (blue arrows) and sinks (red arrows) of phosphorus species on the early Earth. See text for details*.*

On the modern Earth, acidic weathering of apatite in crustal rocks supplies most of the P(V) to the ocean while sea-floor weathering, authigenic apatite and carbonate precipitation, iron oxide precipitation and organic P burial act as sinks (figure 2) [43]. The balance between the source and the sinks ultimately controls the availability of P(V) in modern marine systems. In the Archaean, apatite weathering on land might have been five times more intense compared with modern oxidative weathering owing to higher concentration of CO_2_ in the atmosphere [44]. However, on the prebiotic world, the majority of Earth’s surface may have been covered with water, leading to a less significant supply of land-derived P(V) into the open ocean. Instead, meteorites, sea-floor weathering and most likely hydrothermal vents acted as sources of P(V) on the prebiotic Earth [45,46]. Meteorites, in particular, could have provided soluble P minerals such as schreibersite ((Fe,Ni)3P), hydrated ammonium–magnesium phosphate and whitlockite [45,47]. At that time, the major sink of P(V) would have been precipitation of authigenic apatite, vivianite and carbonate precipitation and adsorption onto green-rust [48,49]. Organic P burial and Fe-oxide-induced removal would have been limited in the absence of life and O_2_.

Vivianite solubility experiments, modelling and carbonate-hosted P(V) data suggest that P(V) in the marine environments could have reached tens of micromolar to millimolar concentrations in the early Archaean [48]. Furthermore, alkaline pools on volcanic islands might have offered even higher P(V) concentration facilitated by the precipitation of Ca-carbonates instead of apatite [50]. Hence, there were likely places on the prebiotic Earth that offered high concentrations of dissolved P, solving one part of the ‘P-problem’.

However, the low reactivity with P(V) remains an issue for abiotic formation of biomolecules. Several other hypotheses have, therefore, been invoked to address the ‘P-problem’ for the origin of life, considering that the prebiotic world was highly anoxic and exposed to frequent meteorite impacts compared with modern Earth [45]. In particular, reduced P such as phosphite (${\text{HPO}}_{3}^{2^{-}}$, P(III) with P having an oxidation state of +3), hypophosphite (P(I): P with an oxidation state of +1) and phosphonate (molecules with P–C bonds and P with +3 oxidation state) are more soluble than P(V). For example, P(III) is *ca* 1000 times more soluble than P(V) in natural fluids, including seawater, in the presence of bivalent metals such as Ca, Mg and Fe(II), and kinetically stable in anoxic prebiotic environments. P(III) has also been shown to be more efficient than P(V) in forming organophosphorus compounds [39], and given that P(I) is even more reactive than P(III), and P–C compounds already contain P–C bonds, these reduced P species may act as more efficient phosphorylating agents than P(V).

On the prebiotic Earth, several geological processes could have produced P(III), such as (i) the reduction of P(V) facilitated by concomitant oxidation of Fe^2+^ to Fe^3+^ during diagenesis and metamorphism [51,52], (ii) P(V) reduction during serpentinization [52,53], (iii) lightning-induced reduction of soil-hosted P(V) [54], and (iv) the dissolution of phosphide minerals, such as schreibersite ((Fe,Ni)_3_P), either delivered by meteorites and/or produced in soils during lightning strikes or in contact metamorphic rocks [45,54,55]. Among these processes, (i) and (iv) might have produced P(I), while (iv) might have generated P–C compounds. Serpentinization, meteoric supply and lightning (depending on land-exposure) could have provided reduced P species on a global scale, while contact metamorphism-induced production might have occurred locally near active volcanoes. The major sinks of these reduced P species, in particular their (photochemical and dark) oxidation rates to P(V) in different prebiotic environments remain to be explored, which at present prohibits accurate estimates of reduced P concentrations in prebiotic water bodies.

Another group of P(V) molecules, namely polyphosphates or condensed phosphates, which include pyrophosphate (PPi), triphosphate (PPPi), tetra- and other higher-order phosphates (PPPPi+), and cyclophosphates such as trimetaphosphate (PPPc) and tetrametaphosphate (PPPPc), are more reactive than P(V) and may have helped in phosphorylation processes on the prebiotic Earth. For example, cyclophosphates may act as phosphorylating agents, including of glyceric acid, sugars, amino acids and nucleosides [56–58]. Several geological processes could have produced polyphosphates on prebiotic Earth, such as (i) dry-heating of sodium or ammonium phosphates (e.g. NaH_2_PO_4_/NH_4_H_2_PO_4_) at 80–1200°C [59,60]; (ii) high-temperature (>1200°C) volcanic processes [61]; (iii) aqueous dissolution of metallic phosphide [62]; and (iv) thermal metamorphism of Fe–P-rich sediments [52]. Besides that, several P(V) polymerization routes have been described in the presence of organic compounds and non-aqueous solvents, which might have been important in some locales [58]. Although it has been argued that polyphosphates are more soluble than P(V), most of the data are restricted to simple acids or salts. Detailed studies on the solubility of these species in prebiotic environments are needed. Furthermore, the major sinks of these polyphosphates in the prebiotic environments are largely unknown. Dissolved concentrations of these compounds can, therefore, currently not be estimated.

## 4. Metal mobilization

Many transition metals play essential roles in modern metabolisms, where they have structural, metabolic or catalytic cellular functions [63]. The solubility and catalytic properties of metals during the Hadean and Archaean likely influenced the origin and evolution of early (proto-)metabolic networks and metalloenzymes. For example, the biogeochemical nitrogen cycle utilizes metalloenzymes at every step [64]. This includes biological nitrogen fixation—an ancient metabolism that likely evolved during the Archaean and relies on both Fe and Mo metal cofactors [65,66]. Another example is methanogenesis via the acetyl-CoA pathway, thought to be one of the oldest CO_2_-fixation pathways, which requires Fe, W, Se, Zn, Co, Ni and Mn [67]. Seawater on the early Earth was likely reducing and more acidic (pH 6.5−7) than today [68,69], with implications for metal solubility. The two major point-sources of metals to the environment, prior to the advent of oxidative weathering, were likely deep-sea hydrothermal vents and terrestrial hot spring systems.

Sea-floor hydrothermal vents have long been proposed as major players in prebiotic chemistry [70]. They are expressions of submarine volcanism and dominated the marine metal budgets on the early Earth [71]. They exhibit a range of physiochemical conditions, including low-temperature alkaline hydrothermal vents created by serpentinization of (ultra-)mafic igneous rocks and acidic high-temperature vents fuelled by magmatic heat [72]. High-temperature vents disperse high concentrations of many catalytic metals, including Fe, Mn, Cu, Zn, Ni, Co and Mo, which are found in many ancient metabolisms [1,2,73–75] and may date back to prebiotic reactions. In contrast, low-temperature alkaline vents are relatively metal-poor, but in combination with magmatic heat sources may have offered ideal sites for Fischer–Tropsch-type reactions to convert CO_2_ into hydrocarbon molecules (as well as N_2_ conversion to ammonium, see above), catalysed by FeNiS [76]. Concentrations of many transition metals in the early ocean were once thought to be several orders of magnitude lower than today owing to scavenging by sulfide [77]; however, empirical data suggest Cu and Zn concentrations were only slightly lower [71,74] and Ni and Co concentrations were higher than today [1,2] (table 1, see table S5 for references). Fe and Mn levels were also likely much higher than today, owing to intense hydrothermal input. Molybdenum, however, probably remained relatively insoluble [79], despite a demonstrated hydrothermal influx [75] (table 1).

**Table 1.** Average dissolved concentrations of transition metals within modern and Archaean oceans based on published literature (see electronic supplementary material, table S5 for references) alongside average concentrations within modern hot springs, based on averages of values in online supplementary material, table S1 [78].

metal	units	modern ocean	Archaean ocean	hot spring average (pH < 6)	hot spring average (pH > 6)
Co	μg l^−1^	0.001	0.002	3.340	0.8
Cu	μg l^−1^	0.150	0.013	39.2	9.4
Fe	μg l^−1^	0.030	5500	11 412	213
Mn	μg l^−1^	0.020	275	467	247
Mo	μg l^−1^	9.900	0.300	4.9	25
Ni	μg l^−1^	0.480	23.00	24.4	2.0
V	μg l^−1^	1.800	unknown	41.4	1.9
Zn	μg l^−1^	0.350	0.060	221.3	67

Less understood are terrestrial hot springs in the context of metal sources for prebiotic chemistry. Terrestrial hot springs driven by subaerial volcanism fall into four categories, depending on their fluid chemistry and mineralogy: alkaline–chloride fluids that contain high levels of silica and precipitate silica sinter deposits; acid–sulfate springs where volcanic H_2_S oxidizes to strongly weathering sulfuric acid; iron-rich springs resulting in precipitation of iron oxide minerals; and bicarbonate-rich fluids, which pass through carbonate rocks and precipitate calcium carbonate at the surface [80]. Host bedrock and secondary mineralogy combined with deep and surficial geothermal processes that collectively influence pH, temperature and dissolved chemistry can create a variety of localized, interconnected and often transient geochemical niches. This creates heterogeneous dissolution of catalytic metals [79,81], exploited by hot spring microbial communities on the modern Earth [82–84]. Likewise, impact-driven hydrothermal systems within both terrestrial and submarine settings would contribute to localized mobilization of metals relevant to prebiotic chemistry [85].

**Figure 3.**
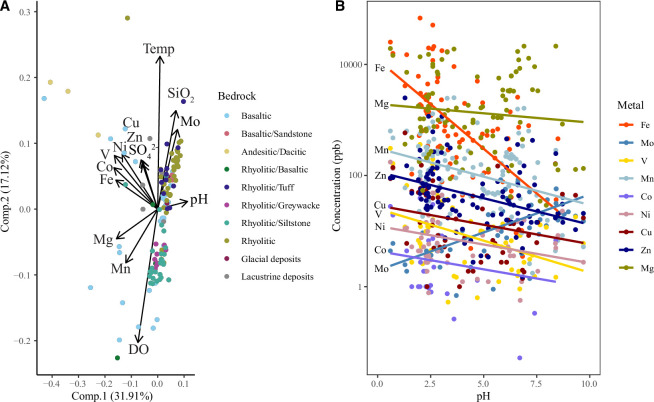
(A) Plot of a principal component analysis (PCA) of hot spring physicochemical parameters and dissolved metal abundances, with datapoints coloured by the lithology of their host bedrock (see legend). References and data can be found on the British Geological Survey repository [78]. Principal components 1 and 2 (Comp. 1 and Comp. 2) account for 49.03% of variance in the data. Temp, temperature; DO, dissolved oxygen. (B) Plot of dissolved logarithmic metal abundances against pH of modern hot spring systems, with lines of regression fitted to data. These plots were generated in RStudio [86] and modified in Adobe Illustrator (2025)*.*

To refine knowledge about the metal budget of terrestrial hot springs, we collated geochemical data from multiple modern analogues (online supplementary material [78, tbl. S1]) and applied a principal component analysis (PCA) to examine correlations between dissolved metal abundance and physicochemical parameters such as pH, dissolved oxygen, temperature and bedrock (figure 3A). We also produced multiple general linear models to test the statistical significance of any correlations (script and models available on GitHub [86]). Results of the PCA suggest that pH accounts for 31.91% of variability in dissolved metal abundances, while temperature accounts for 17.12%. The concentration of each metal across the pH range of this hot spring dataset is shown in figure 3B. Most metals show decreasing abundance with increasing pH, except Mo, which instead is found in higher abundances within more alkaline waters (model6, *p*‐value = 0.062, adj *R*^2^ = 0.73 [86]), agreeing with previous studies [87] (table 1). Neutral–alkaline (pH >6) hot springs may thus have created Mo-enriched niches, in comparison with the Mo-depleted early ocean (table 1). Our model included interactions between Mo and both temperature and dissolved oxygen (DO), as these variables both influence Mo (DO accounts for 12.94% of variability). While oxygenated high-temperature waters favour solubility of Mo [87], this is not seen in the PCA plot owing to the decreasing solubility of O_2_ within high-temperature waters; interactions between Mo and temperature or DO were not significant in statistical models. Additionally, the correlation between Mo and pH was found to be statistically insignificant when interactions between Mo and SiO_2_ were included in linear models (model9, *p*‐value = 0.28, adj *R*^2^ = 0.74 [86]). This is because bedrock also influences metal supply, as is seen in figure 3A, although no statistically significant relationship was seen between Mo and SiO_2_ (model9, *p*‐value = 0.83, adj *R*^2^ = 0.74 [86]). Silica-rich rhyolitic bedrock is relatively enriched in Mo, while Fe, Mg, Mn, Cu, Ni, V and Co are found in highest abundances within basaltic bedrock [78, tbl. S3]. This highlights the complexity of factors that influence both metal supply and each other, meaning that simple correlations between two variables are difficult to examine statistically.

The solubility relationships between these metals and their environments depend on their geochemical classification. Chalcophile elements (Co, Cu, Mo, Ni and Zn) are only soluble within sulfide-poor waters, as they form insoluble metal sulfide precipitates [88], although solubility increases with temperature [74]. These metals thus tend to be found in acidic, oxidizing conditions alongside ${\text{SO}}_{4}^{2-}$ (figure 3). Siderophile metals (Co, Fe, Mn, Ni and V) are found alongside Fe as expected. One exception is Mo, which exhibits both siderophile and chalcophile characteristics, depending on pressure and temperature, as well as its tendency to form insoluble oxyhydroxides with Mn/Fe in oxic conditions [79,89]. Thus, it is typically found in systems with low Mn (figure 3) or Fe today. On a prebiotic Earth, in the absence of O_2_, the solubility of chalcophile metals in hot springs may thus have been overall lower than today owing to sulfide scavenging (table 1); however, elevated temperature would have offset this effect to some extent. Overall, the solubility of Mo may have been highest in settings with high pH and elevated temperature. Acidic conditions may have maintained a high supply of siderophile metals.

Our compilation highlights the complex variety of factors that influence metal mobility in both modern and early Earth waters. Similar to today, metal supply on the prebiotic Earth likely varied with local conditions and displayed numerous niches of metal enrichment both in terrestrial hot springs and near deep-sea vents that facilitated metal-catalysed reactions, akin to metallo-enzymes. For instance, the MoFe nitrogenase enzyme is thought to have evolved by at least 3 Ga [90]. Under prebiotic or early biotic conditions, this enzyme may have evolved in alkaline geothermal systems on land or in the vicinity of hydrothermal vents in the deep sea to gain access to Mo, which was otherwise unavailable within early oceans (table 1). Many other metalloenzymes also require metals that are found in distinct geochemical niches, supporting the idea that prebiotic chemistry may have benefited from physicochemical gradients and environmental diversity. Importantly, however, the biochemical utility of an element is not only dictated by availability but also by thermodynamic efficiency. For example, although Mo was less prevalent than other metals, the MoFe-dependent nitrogenases evolved earlier than FeFe- and VFe-varieties [66], likely because the MoFe enzyme requires less cellular energy to catalyse nitrogen fixation [91]. Hence the chemical properties of elements must be considered in order to draw inferences about their importance and effects on prebiotic chemistry on Earth and other planetary bodies.

## 5. Summary and outlook

In summary, there would have been numerous sources of phosphorus, nitrogen and reactive metals on the Hadean/Archaean Earth, but our review highlights the importance of submarine and terrestrial volcanism for driving prebiotic processes and maintaining early biological communities.

Conversion of atmospheric N_2_ into NH_4_^+^ can be catalysed abiotically through lightning or in hydrothermal vent conditions with metallic minerals [12,14–16,19–26]. The mineral-catalysed reduction is favoured at elevated pressures [22] and thus is unlikely within terrestrial hot spring systems. However, rock weathering may provide higher levels of NH_4_^+^ to these systems if they are hosted within siliceous igneous rocks such as in parts of modern Iceland [28,29]. Therefore, hot springs could have hosted key prebiotic reactions and early microbial ecosystems, supported by rock weathering as well as sporadic lightning input [23]. NH_4_^+^ is the most efficient form of nitrogen for cell uptake [89,92] and would be chemically stable within anoxic, reducing waters such as the Hadean oceans, hydrothermal vents and terrestrial hot springs [93] (figure 1).

Phosphorus is often unavailable to the biosphere as it is commonly trapped into insoluble mineral precipitates [40], and weathering processes would have been much slower on the early Earth owing to smaller land masses [44]. However, P is highly soluble within alkaline waters and alkaline hot springs, as well as hydrothermal vents [50]. In addition to dissolution of P, the redox state of P species is important for prebiotic reactions as P(V) has very low reactivity and would be inefficient for the spontaneous formation of phosphorylated biomolecules [40]. Reduction of P(V) to more reactive species can occur during lighting strikes, serpentinization and meteoritic impacts, while localized volcanism and metamorphic heating may have also facilitated reduction of P(V) via Fe^2+^ oxidation [45,51–55]. Other more reactive P species such as polyphosphates could have also been created within high-temperature geothermal systems [52,59–61]. These reduced P species are more soluble than P(V) and thus would have been more favourable for prebiotic reactions.

Previous research has shown that high-temperature hydrothermal vents are capable of providing high levels of transition metals, including Mo, an essential metal used in multiple biological nitrogen pathways [1,2,73–75]. Our study also looked at the availability of trace metals in modern hot spring systems and found that pH, temperature, DO levels and bedrock all influence metal availability in sequentially decreasing proportions (figure 3). High-temperature, acidic, basaltic-hosted springs favour siderophile metals, while high-temperature, alkaline and siliceous springs favour dissolution of Mo. The chemical characteristics of transition metals impact their availability and stability, as chalcophilic metals such as Mo will precipitate out of solution in euxinic conditions [87]. As highlighted in the PCA plot, this effect may be lessened in high-temperature environments such as hydrothermal vents or hot springs that encourage dissolution [74]. Geothermal systems thus provide ideal conditions for dissolution of catalytic metals, albeit with high spatial heterogeneity created by variations in pH and temperature.

The suite of elements discussed above is found in every form of life discovered thus far on Earth, and it is conceivable that life on other planets would use similar chemical elements. This is especially important when considering the habitability of planets similar to our own, such as Mars. The Noachian period of Mars’s history (4.1–3.7 Ga) is considered to be its most habitable era owing to the presence of an active hydrological cycle [94]. Mars rover missions have identified deposits of nitrogen, phosphorus and many transition metals *in situ* on the surface of Mars [95–99]. The *Spirit* rover’s alpha-particle X-ray spectrometer measured high levels of Zn in Gusev Crater along with Ni, Fe, Mn and Mg [95]. These metals, along with unusually high levels of Cu, were also detected by the *Curiosity* rover in Gale Crater [99]. V, Mo and Co have yet to be detected *in situ* but have been measured in multiple Martian meteorites [100,101]. The concentration of metals in Martian rocks and meteorites is similar to the basaltic compositions seen on Earth [78, tbl. S2 and S3] and suggests that Mo would have also been in low abundance within Martian environments, resulting in both planets experiencing similar geochemical prebiotic bottlenecks. Evidence of both hydrothermal vents and terrestrial hot springs has been documented on the surface of Mars within Noachian-age deposits [102–104], which may have similarly mobilized these elements into the environment. Geothermal environments such as terrestrial hot springs, volcanic lakes or hydrothermal vents may, therefore, have provided all of the essential elements for the origin of life not just on the early Earth but perhaps also Noachian Mars and beyond.

## Data Availability

The data used to generate figure 3 are available in table S1 of the supplementary data uploaded to the British Geological Survey (BGS) repository [78, tbl. S1]. This includes collated literature data of hot spring water chemistry, published data by Galloway et al. [105] and additional unpublished chemical data not included elsewhere, along with methods for dissolved ion analyses. This dataset also includes collated literature data of metal abundances in differing bedrocks on Earth and Mars [78, tbl. S2], and average metal abundances of each bedrock type [78, tbl. S3] along with references for all literature-sourced data [78, tbl. S4]. The R script used to perform and plot the principal components analysis in figure 3 as well as general linear models are available on GitHub [86]. Values included in table 1 and their corresponding literature references can be found in [78, tbl. S1, S4 and S5]. Supplementary material is available online [106].
